# Predicting first time depression onset in pregnancy: applying machine learning methods to patient-reported data

**DOI:** 10.1007/s00737-024-01474-w

**Published:** 2024-05-22

**Authors:** Tamar Krishnamurti, Samantha Rodriguez, Bryan Wilder, Priya Gopalan, Hyagriv N. Simhan

**Affiliations:** 1 Division of General Internal Medicine, University of Pittsburgh, 230 McKee Pl, Suite 600, Pittsburgh, PA 15213, USA; 2 Machine Learning Department, Carnegie Mellon University, Pittsburgh, PA 15213, USA; 3 UPMC Western Psychiatric Hospital, Pittsburgh, PA 15213, USA; 4 Department of OB-GYN and Reproductive Sciences, University of Pittsburgh, Pittsburgh, PA 15213, USA

**Keywords:** Depression, Pregnancy, Risk prediction, Machine learning, Mhealth, Social determinants of health

## Abstract

**Purpose:**

To develop a machine learning algorithm, using patient-reported data from early pregnancy, to predict later onset of first time moderate-to-severe depression.

**Methods:**

A sample of 944 U.S. patient participants from a larger longitudinal observational cohortused a prenatal support mobile app from September 2019 to April 2022. Participants self-reported clinical and social risk factors during first trimester initiation of app use and completed voluntary depression screenings in each trimester. Several machine learning algorithms were applied to self-reported data, including a novel algorithm for causal discovery. Training and test datasets were built from a randomized 80/20 data split. Models were evaluated on their predictive accuracy and their simplicity (i.e., fewest variables required for prediction).

**Results:**

Among participants, 78% identified as white with an average age of 30 [IQR 26–34]; 61% had income ≥ $50,000; 70% had a college degree or higher; and 49% were nulliparous. All models accurately predicted first time moderate-severe depression using first trimester baseline data (AUC 0.74–0.89, sensitivity 0.35–0.81, specificity 0.78–0.95). Several predictors were common across models, including anxiety history, partnered status, psychosocial factors, and pregnancy-specific stressors. The optimal model used only 14 (26%) of the possible variables and had excellent accuracy (AUC = 0.89, sensitivity = 0.81, specificity = 0.83). When food insecurity reports were included among a subset of participants, demographics, including race and income, dropped out and the model became more accurate (AUC = 0.93) and simpler (9 variables).

**Conclusion:**

A relatively small amount of self-report data produced a highly predictive model of first time depression among pregnant individuals.

## Introduction

Perinatal depression is one of the most prevalent pregnancy complications in the US. Approximately 15% of individuals report depressive symptoms at some point in their pregnancy (Dietz et al. 2007), with instances of major depression occurring among roughly 12% of pregnancies (Le Strat et al. 2011) and in the postpartum period (Evans et al. 2001). Depression rates are lowest in the first trimester and tend to spike in the second trimester and again postpartum (Yu et al. 2020). Early adulthood, when many first pregnancies occur, is a time of particular vulnerability to depression onset (Rohde et al. 2005). Perinatal depression is associated with adverse outcomes for mother and infant (Chung et al. 2001), including preterm delivery (Grigoriadis et al. 2013), delayed infant/child development (Madigan et al. 2018), decreased quality of life (Lagadec et al. 2018), and poor maternal-child attachment (Santoro and Peabody 2010).

The American College of Obstetricians and Gynecologists (ACOG Committee Opinion No. 757 Summary: Screening for Perinatal Depression 2018), the US Preventive Services Taskforce (U. S. Preventive Services Task Force et al. 2019), and other professional organizations recommend early and routine perinatal screening for depression risk. Established perinatal depression screening tools, such as the Edinburgh Postnatal Depression Scale (EPDS) (Cox et al. 1987) and PHQ-9 (Spitzer et al. 1999), are effective at detecting current depressive symptoms. However, these screening tools are inconsistently administered during the course of pregnancy, with common practice being to screen once during early pregnancy and not again until the postpartum period (Long et al. 2020; Sidebottom et al. 2021). As such, many instances of active depression may be missed during routine care. Moreover, these screening tools are not intended for the identification of future depression risk (Cox 2017; Kroenke et al. 2001). Both for prevention and intervention, there is a need for screeners that can be administered early in pregnancy to identify those who are likely to develop new depressive symptoms at a future stage of pregnancy.

Studies of perinatal depression trajectories find heterogeneity in both symptoms and the risk factors associated with the timing and duration of those symptoms (Ahmed et al. 2018; Bayrampour et al. 2016; Mora et al. 2009). Depression symptoms in pregnancy are consistently associated with postpartum depression occurrence. History of depression prior to pregnancy is, in turn, one of the strongest predictors of depression during pregnancy (Guintivano et al. 2018a; Lancaster et al. 2010; O’Hara and Swain 1996). Consequently, depression history is an extremely useful cue for identifying those who may experience recurrent depression part way through their pregnancy and into the postpartum period. However, approximately 50% of individuals experiencing depression in pregnancy report no prior depression history, necessitating use of other risk cues for those individuals (McCall-Hosenfeld et al. 2016). Other documented perinatal depression risk factors that can be measured in early pregnancy include having an unplanned pregnancy (Abajobir et al. 2016; Cheng et al. 2009; Orr et al. 2008), previous pregnancy complications (Blackmore et al. 2011; Egan et al. 2017; Silverman et al. 2017; Zhang et al. 2020), other concurrent mental health conditions, such as anxiety, (Korja et al. 2018; Putnam et al. 2017), stressful life events (Guintivano et al. 2018b; Nunes and Phipps 2013; Qobadi et al. 2016), and health-related social needs, including a lack of social or financial support (O’Hara and Swain 1996; Robertson et al. 2004). Still, little is known about how to use these risk factors concurrently to identify who is likely to experience first time depression in their pregnancy.

As the field of perinatal mental health moves towards precision medicine, machine learning can play a powerful role in shaping care delivery and risk phenotyping, including digital phenotyping, offering an opportunity for earlier intervention (Hurwitz et al. 2023; Osborne et al. 2019; Torous et al. 2015). Machine learning algorithms have shown promise in their ability to predict maternal depression (Cellini et al. 2022; Zhong et al. 2022). A 2021 review by Ramakrishnan et al. found that the majority of perinatal depression models have focused on prediction of depression in the postpartum period and include individuals with a prior history of depression (Ramakrishnan et al. 2021; Robertson et al. 2004). Other review studies show that perinatal depression algorithms have largely been built on data mined from big data sources, such as electronic health records or social media data (Zhong et al. 2022). While these models perform well, they may fail to incorporate the kinds of contextual social factors that influence mental health outcomes, and which can only be elicited through patient self-report, such as stressful life events. Often these machine learning models are considered to be a ‘black box,’ using methods that obscure which data is being used or how the algorithm actually works with the data to identify risk factors. When machine learning models are difficult – if not impossible – to interpret, they pose a challenge to providers that are trying to understand the underlying triggers of depression risk (Rudin 2019; Schmude et al. 2023). Moreover, such ‘black box’ algorithms cannot be easily translated into the kind of screening tool that can be administered directly to a patient. As such, algorithms implemented into clinical workflows without process transparency may risk non-engagement from healthcare providers (Cutillo et al. 2020; Kelly et al. 2019; Liefgreen et al. 2023; MacKay et al. 2023). Developing algorithms for new depression onset in pregnancy that include self-reported data on social or contextual risk factors, while remaining simple to understand and relevant to the clinicians managing patients, is a critical need in healthcare.

In this secondary analysis of patient-reported data collected through a prenatal smartphone app, we applied machine learning methods to develop an algorithmic model from data collected early in pregnancy (during first trimester app-use) to predict subsequent onset of first time depression in pregnancy. To identify a predictive model that could ultimately be translated into a patient-facing screener in the early prenatal care workflow, we compared a set of machine learning methods chosen for their ability to reduce the number of variables required in the model and their ease of interpretation. All models were used to predict new moderate-to-severe depressive symptoms among individuals with no prior depression history.

## Methods

### Study sample

Data was collected using the MyHealthyPregnancy^™^ (MHP) smartphone app from September 2019 to April 2022. Patients of the University of Pittsburgh Medical Center (UPMC) healthcare system were prescribed MHP during their first prenatal visit (8 +/− 2 weeks’ gestation) as part of a quality improvement initiative to supplement routine prenatal care with a digital support (ethics board approval project number: 1684). Patients were eligible for inclusion in this study if they were 18 + years of age, had no self-reported history of depression, completed a first trimester screener for potential risk factors and voluntarily completed at least one subsequent EPDS through the MHP app. All study procedures were approved by the University of Pittsburgh’s Institutional Review Board (STUDY2207002). Data analyses were performed in 2023.

### App-collected patient-report data

MyHealthyPregnancy (MHP) is an evidence-based mHealth tool for use during pregnancy (Bohnhoff et al. 2021; Castillo et al. 2022a, b; Krishnamurti et al. 2017, 2021, 2022a, b, c; Mora et al. 2020). It provides education organized by the user’s week of gestation and connection to relevant resources within and outside the healthcare system. MHP also offers pregnancy tracking tools, such as a daily assessment of mood and symptoms, a diary to document the user’s pregnancy experiences, a fetal movement counter and contraction timer, and routine screenings for psychosocial risks. In addition, the app uses patient-entered data to alert providers to possible risks during pregnancy.

Upon first use of MHP, patients were asked a baseline (early first trimester) questionnaire consisting of demographics (e.g. “*Which best describes your household income in the past 12 months?*”), medical history (e.g., “*Is this your first pregnancy?*”), and psychosocial questions (e.g., “*Have you recently been feeling unusually stressed about going into labor and giving birth?*”). Patients could also volunteer self-reported experiences of health-related social needs (e.g., “*Within the last 12 months, have you worried you would run out of food, or did you run out of food, before being able to buy more?*”) throughout their pregnancy. All patients were subsequently prompted once a trimester on the app’s home screen to use the app-embedded EPDS for depression self-screening. From July 2021 onwards, the EPDS was available in the app for voluntary self-screening for depression symptoms at any point during the pregnancy. A cutoff EPDS score of 13 or more (moderate to severe depression risk) reported at any time during the pregnancy, but after the initial early first-trimester baseline questionnaire administration, was used to define new perinatal depression symptom onset (Cox et al. 1987; Hewitt et al. 2009; Levis et al. 2020).

### Machine learning

Six machine learning models were trained and applied to patient-provided app data shared in the early first trimester baseline questionnaire to predict instances of new-onset perinatal depression symptoms (EPDS ≥ 13) later in pregnancy among participants with no self-reported history of depression. Three models were chosen to prioritize a smaller set of variables: Least Absolute Shrinkage and Selection Operator (LASSO) (Tibshirani 1996), Forward Stepwise Selection (FSS) (Kutner et al. 2005), and Shallow Decision Trees (SDT) (Breiman 2017; Ripley 2007). Two models were chosen to ensure that potential nonlinear relationships, which might not otherwise have been identified in the other models, were captured: Random-Forest (Breiman 2001) and Extreme Gradient Boosting (XGBoost) (Chen and Guestrin 2016). Lastly, a novel method, a nonparametric graph learning approach (Stable P-C) (Colombo and Maathuis 2012; Spirtes et al. 2000) using Kernel-based Conditional Independence (Zhang et al. 2012) (PC-KCI) (Mesner et al. 2019), was chosen to capture potential non-linear relationships while also using a smaller set of variables. All models allow for simple, interpretable output.

Each model was built iteratively, with data being randomly split into 80% for training, withholding 20% for testing, which is aligned with best practice recommendations (Gholamy et al. 2018). Missing data was handled using the dropout method, so only participants with complete data were included in the modeling. As expected, the number of participants in the study cohort who experienced first time depression in their pregnancy was smaller than the number of those who did not experience depression, leading to an unbalanced dataset. To address this, we balanced the training data using random oversampling from among those with first time moderate-severe depression symptoms. Five-fold cross-validation was used to determine which features were retained in each model within the training set. The held-out test data was then used to assess the predictive accuracy of those models. This approach allowed us to develop accurate models while increasing the generalizability of results to future data (Preis et al. 2022).

### Evaluation

Models were evaluated for accuracy (i.e. how well the model distinguishes depression)on the test set using AUC values for predicting moderate-severe (EPDS ≥ 13) depression symptoms. Confidence intervals for the AUC values were calculated using 2,000 stratified bootstrap replicates. To accurately identify at least 80% of cases with first time moderate-severe depression in pregnancy, we set a sensitivity (i.e. the probability of identifying an individual as ‘depressed’ if they have depression) and specificity (i.e. the probability of a identifying an individual as ‘not-depressed’, if they do not have depression) threshold of at least 0.80 using the highest performing model. Consistent with existing literature, sensitivity and specificity were determined with a predicted value threshold of 11 (Alvarado-Esquivel et al. 2006; Horáková et al. 2022; Levis et al. 2020; Teissèdre and Chabrol 2004). Diagnostic Odds Ratios (DORs) were used to measure a model’s ability to discriminate between positive and negative cases at the sensitivity/specificity threshold, with a higher value indicating more successful discrimination. To assess the average point-wise difference between actual and predicted EPDS scores, root mean squared error (RMSE) was calculated for each machine learning model. We considered the number of risk variables retained in each model – or the number of variables required for prediction – to be a measure of the accuracy (AUC) and simplicity for were compared for all six models. DeLong’s test was used for determining statistical differences in AUC values between compared models.

The novel PC-KCI algorithm was implemented using Python v3.10.7. All other modeling and analyses were conducted using R v4.2.2.

## Results

### Demographics

During the study period, a sample of 944 (from a cohort of 5,223) patients using MHP were eligible for study inclusion. Median age was 30 years [IQR 26–34], 70% had at least a college degree, the majority self-identified as white (77.8%), and 61% had a yearly household income of at least $50,000. Population characteristics are shown in Table 1, including differences in baseline characteristics between those with and without new onset depression in the sample.

**Fig. 1:**
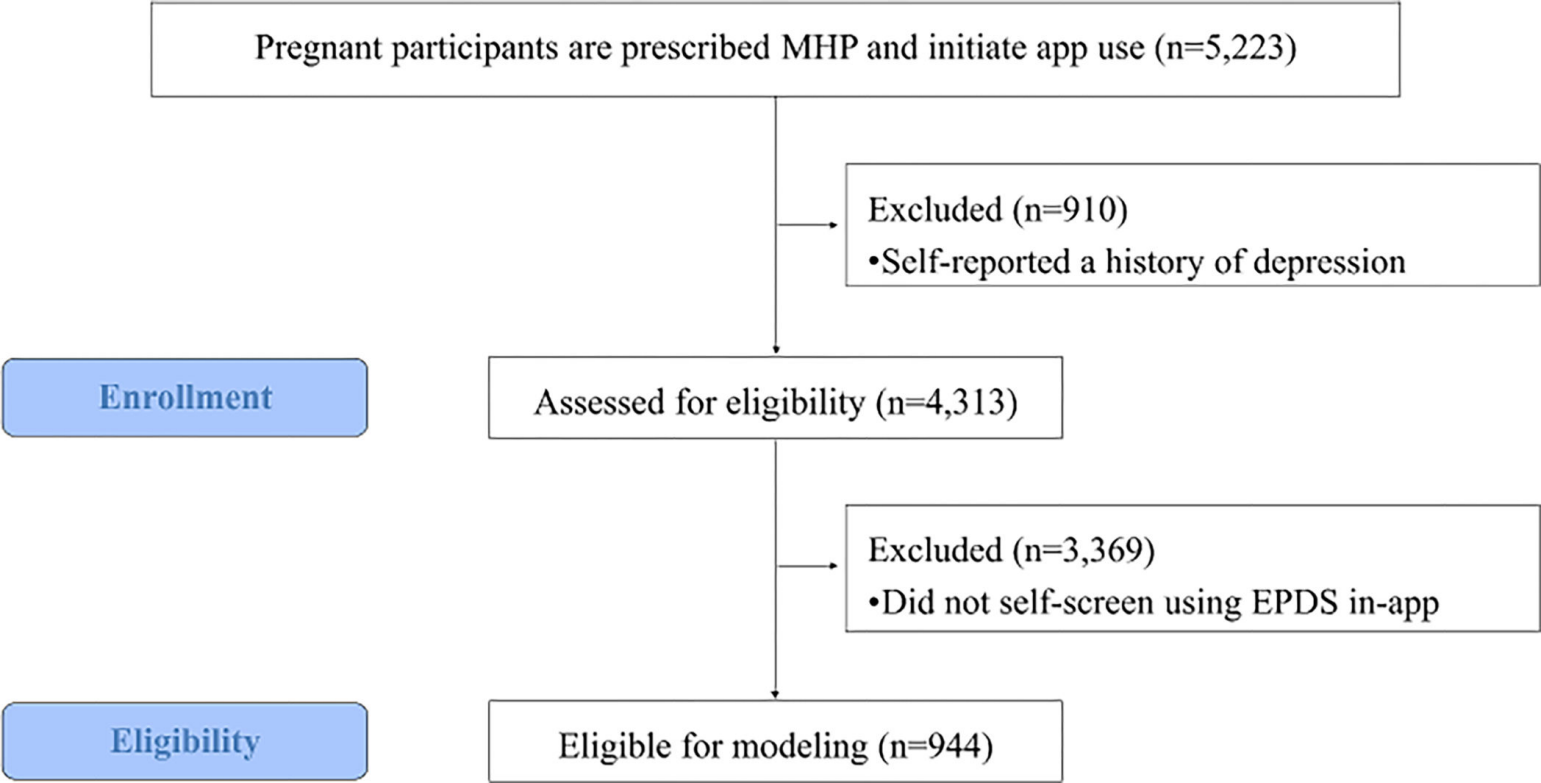
STROBE flow diagram of enrollment in study and eligibility criteria for modeling

All patients using the MHP app at the time of the study completed baseline clinical and psychosocial risk questions upon initiation of app use. Of these, 4,313 (82.6%) reported having no history of depression. Of the individuals with no depression history, 944 (18.1%) also self-screened model’s simplicity for clinical utility. The model’s predictive for depression throughout their pregnancy^1^ and were eligible for inclusion in the study. A subset of 603 of the 944 eligible participants also self-reported their experiences of health-related social needs prior to completing a depression screener and were included in a sub-analysis to understand the additional role of social needs in predicting first time depression onset. Figure 1 shows the data collection flow from initiation of app use to model inclusion.

Of the patients included in our modeling, 110 (11.7%) met the threshold cut-off for moderate-to-severe depression symptoms (EPDS ≥ 13). Patients were more likely to be eligible for inclusion (no reported history of depression and self-screened using EPDS) if they identified as non-white (OR = 1.22, *p* = 0.03) or were over the age of 35 (OR = 1.22, *p* = 0.03). They were less likely to be eligible if they had a history of anxiety (OR = 0.40, *p* < 0.01). Otherwise, there were no demographic differences between those who were eligible for inclusion and those who were not.

### Model performance

Table 2 shows the comparison of predictive performance among the 6 different machine learning models.

Model performance ranged from an AUC value of 0.74 [Shallow Decision Trees] up to an AUC value of 0.89 [PC-KCI], DORs ranged from 4.18 [Shallow Decision Trees] to 20.36 [PC-KCI], while RMSEs ranged from 3.94 [Random-Forest] to 5.17 [Shallow Decision Trees]. Online Resource 1 shows pairwise comparisons of model AUCs to evaluate meaningful differences in accuracy. The AUC achieved by PC-KCI was significantly higher than all other baseline models.

The number of possible risk variables out of the total number of variables was used as a measure of simplicity. Variable-selection models retained anywhere from 24% [Decision Trees] − 82% [LASSO] of the possible risk variables. Shallow Decision Trees retaining the fewest variables (24% or 13/55). PC-KCI and Shallow Decision Trees were both simpler (i.e., require fewer variables for prediction) than LASSO and Forward Stepwise Selection.

**Fig. 2:**
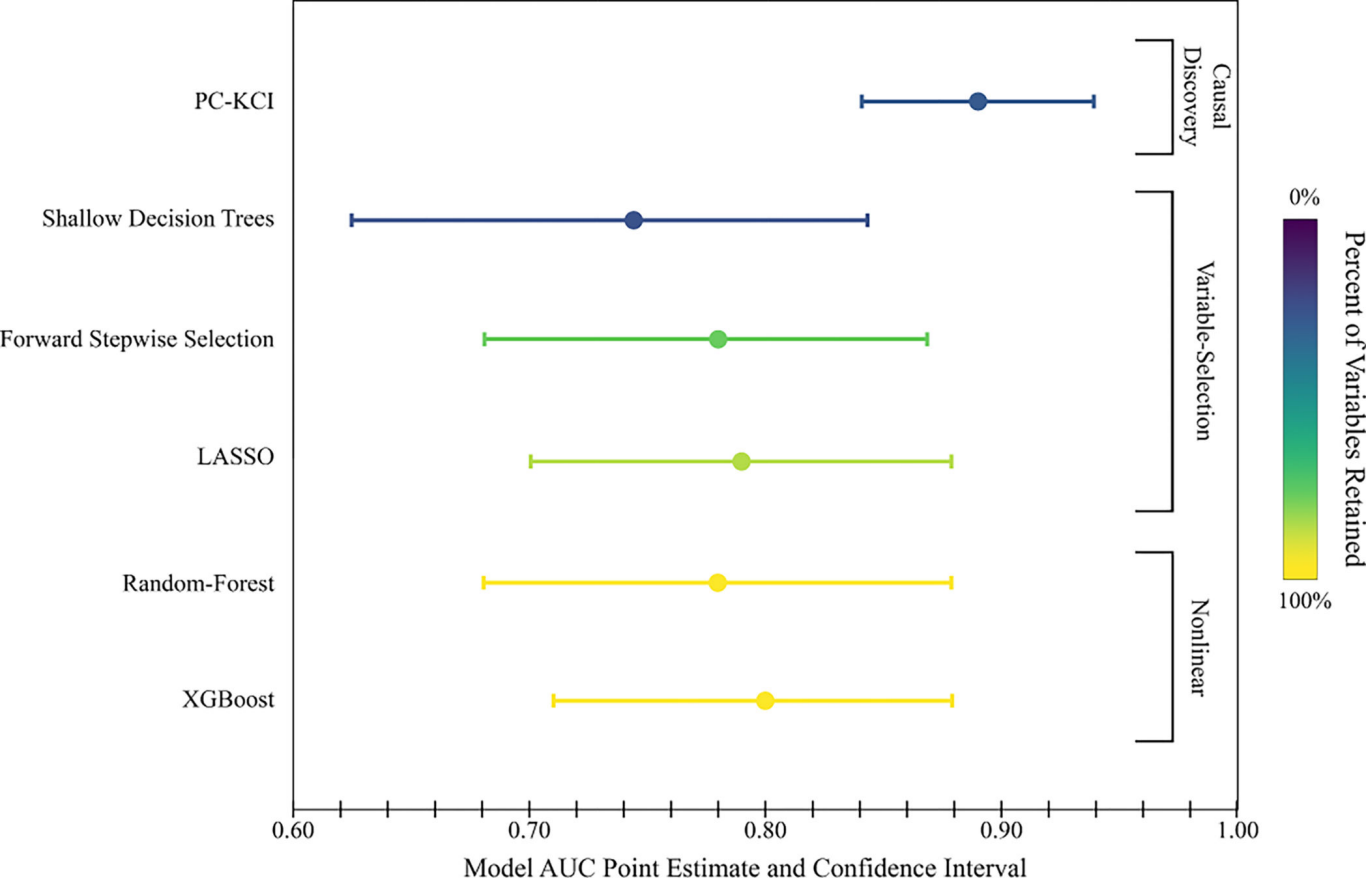
Accuracy (AUC) and simplicity (percent of variables retained) of machine learning models predicting first time depression in pregnancy from patient-reported data

Figure 2 shows a graphical representation of the accuracy and simplicity among the machine learning models. Despite XGBoost having the second highest level of accuracy (AUC = 0.80), neither XGBoost nor Random-Forest achieved high enough accuracy to offset the models’ lack of simplicity. Shallow Decision Trees selected slightly fewer variables than PC-KCI (26% or 14/53). However, PC-KCI was the optimal model overall, maximizing predictive accuracy, while simultaneously minimizing the number of variables required to make that prediction.

### Risk predictors

**Fig. 3:**
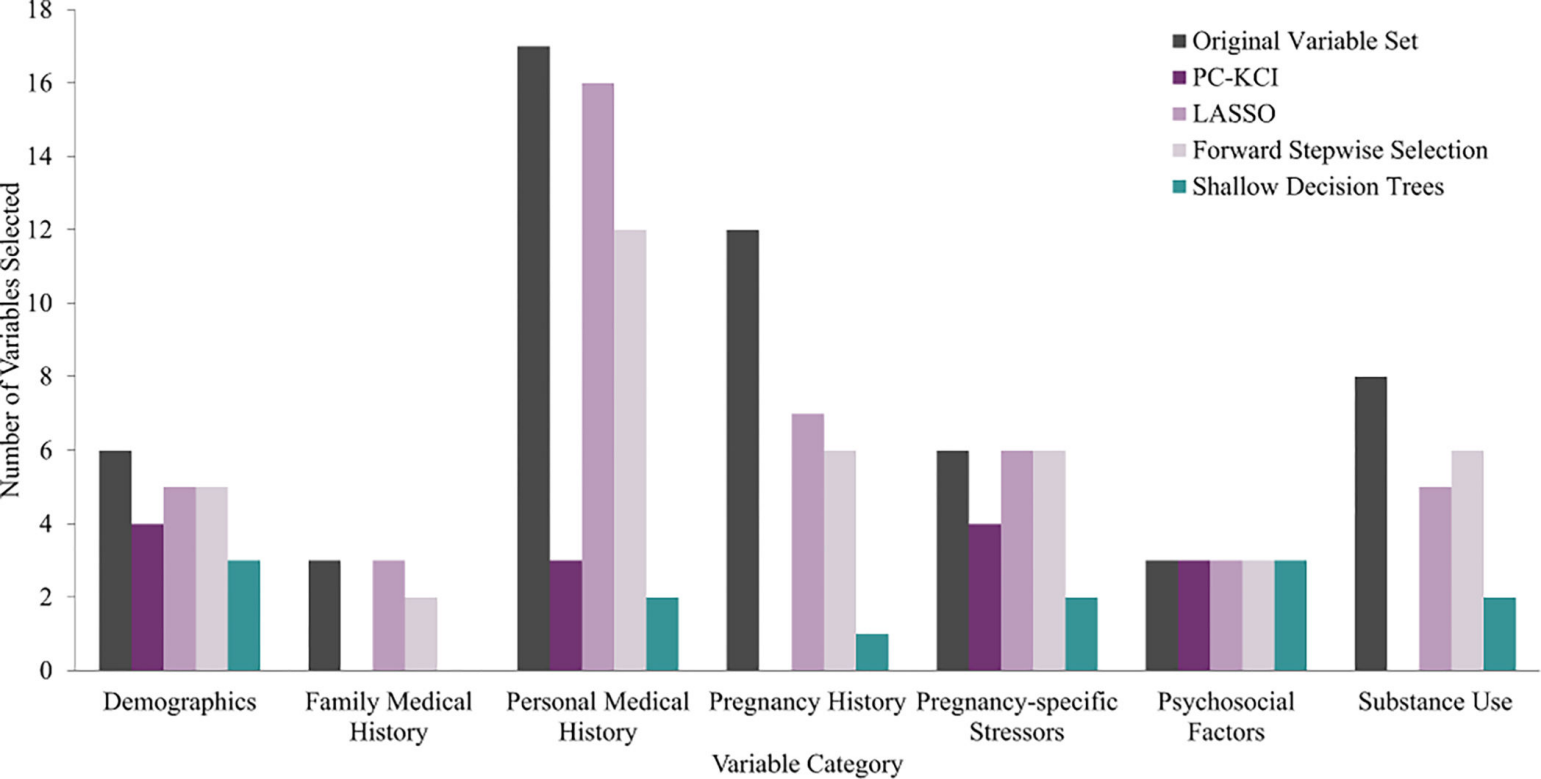
Distribution of selected variables in each variable category by machine learning model

Across all 6 models, variables that were retained from the original 55 variables fell into the broad categories of demographics, family medical history, personal medical history, pregnancy-specific stressors, other psychosocial factors, and substance use. Figure 3 shows the distribution of variables within each broad category that were selected by each of the models.

Online Resource 2 displays those variables retained across the majority of models from each of these categories. Reporting a non-specific but negative change in mood (i.e., feeling “unusually blue”), childhood emotional neglect, stress specific to labor and delivery concerns, and interpersonal relationship stress were all retained as predictive psychosocial variables, regardless of the machine learning model used.

### The relative role of health-related social needs

Online Resource 3 shows the comparison of predictive performance among the machine learning models when health-related social needs were included for that subset of participants that voluntarily reported them prior to completing a depression screener. With the inclusion of health-related social needs, most models became both simpler and more predictive. Almost all models showed improved accuracy when health-related social needs were included in the model, except for two variable-selection models, Forward Stepwise Selection and LASSO. Shallow Decision Trees showed the greatest improvement in accuracy (AUC = 0.92), while remaining simple in structure (only 14 variables required out of 60). PC-KCI and Random-Forest had the highest predictive accuracy, which was not significantly different (*p* = 0.97), although Random Forest achieved a low level of sensitivity (0.20). PC-KCI was, again, the simplest model, only requiring 9 variables for prediction, whereas Random-Forest, by design, requires all variables for prediction. Thus, when including health-related social needs as a risk factor, PC-KCI remained the optimal model (AUC = 0.93, with sensitivity of 0.90, specificity of 0.81, DOR of 38.50, and RMSE of 4.71).

**Fig. 4:**
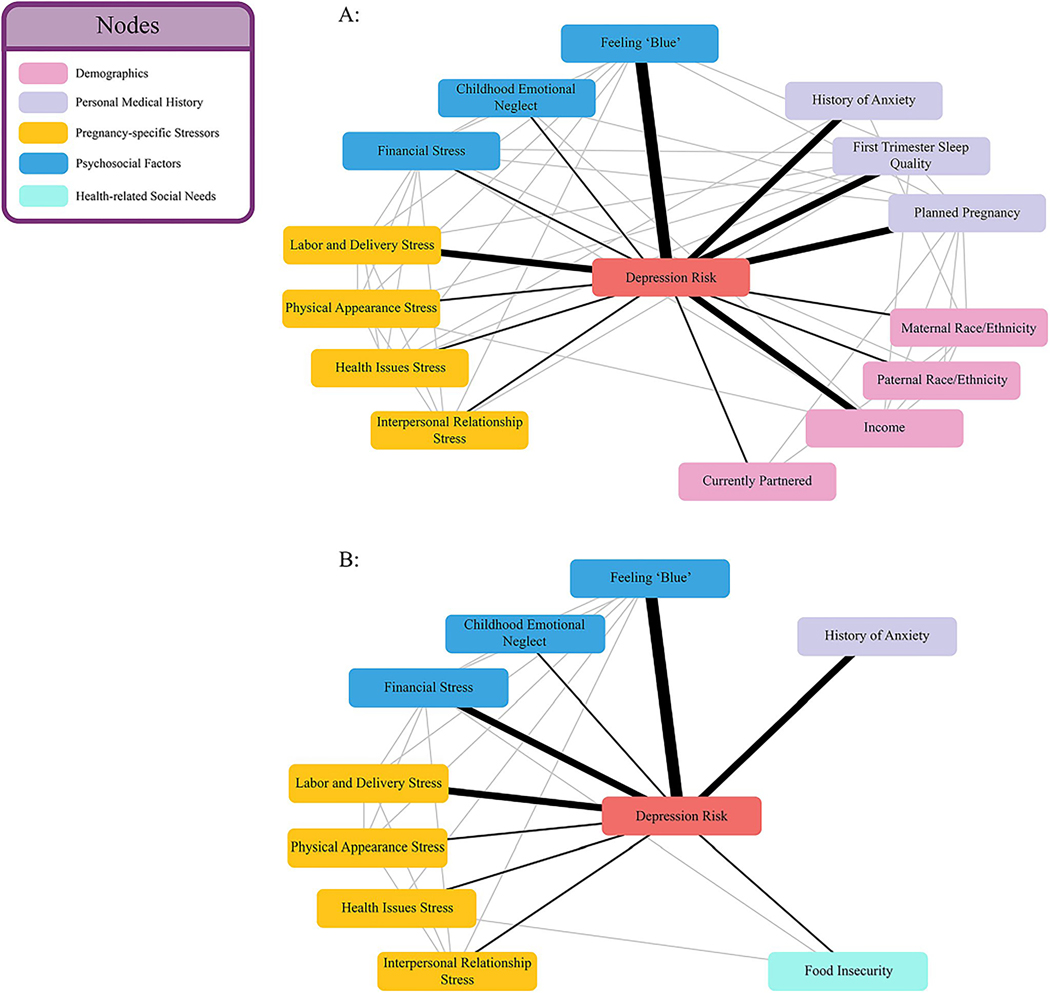
Visualizations of variables retained by the PC-KCI algorithm

Figure 4a and b shows a visualization of the variables retained by the PC-KCI algorithm in the larger sample and in the subset of participants for whom health-related social needs were reported.

Notably, the inclusion of reports of health-related social needs resulted in the retention of food insecurity, as the only health-related social need predictive of depression. Moreover, when food insecurity was included, demographic variables, such as race and income, were dropped from the model. The specific self-report variables included in the PC-KCI model are found in Table 3.

## Discussion

### Principal findings

This study used patient-reported data collected early in pregnancy with a smartphone app to develop several machine learning models for predicting subsequent first time depression onset later in the pregnancy. When comparing approaches that had intentionally been selected for their ability to decrease the number of variables required for predictive accuracy or for their ability to identify nonlinear relationships between risk factors, one algorithm, PC-KCI, was optimal in terms of both accuracy and simplicity. The findings from this study suggest that a small amount of patient-reported data collected early in the first trimester of pregnancy – 14 questions in total – can be used to identify, with substantial accuracy, who will later develop new moderate-to-severe depression onset. Notably, when health-related social needs – specifically, food insecurity – were included for a subset of individuals that reported them prior to first time depression onset, the model became both more predictive and simpler, and no longer included demographic variables. This highlights the critical role that social risk factors play in instances of first time depression, as well as the revealing risks that may be more present among specific demographic groups.

Machine learning methods have been effective at predicting perinatal depression in the prior literature with most published models achieving AUCs of at least 0.70 (e.g., Cellini et al. 2022). The most accurate models tend to be the most complex as they are maximizing accuracy by using all possible data (Kelly et al. 2019). To have practical clinical utility, however, models should be explainable and, ideally, simple enough to implement (MacKay et al. 2023). In previous models of perinatal depression prediction, nonlinear machine learning models tend to perform the best (Andersson et al. 2021; Preis et al. 2022; Shin et al. 2020) and are the most common choice across studies (Zhong et al. 2022); indicating the complexity of the relationship between risk factors and depression. Similarly, in the present analysis, non-linear models performed well in predicting first time depression. However, when additionally considering simplicity for the purpose of clinical utility, only one model was optimal – PC-KCI. This finding highlights the opportunity to consider the selection of algorithms, including graph learning methods like PC-KCI, to prioritize their practical use for clinicians and to glean new insights on the relationships between potential clinical risk factors.

Consistent with existing studies, our optimal model identified factors related to previously documented domains of risk, such as demographic factors (e.g., income), clinical history (e.g., prior anxiety), and experiences of stressful events (Augusto et al. 2020; Preis et al. 2022; Robertson et al. 2004; Yu et al. 2020; Zhong et al. 2022). However, the best-performing model also shed light on pregnancy-specific stressors that might uniquely contribute to first time instances of perinatal depression onset, such as concerns regarding labor and delivery and negative perceptions of the pregnancy affecting physical appearance or future health. The findings also point to the relative strength that underlying, non-specified poor mood may play in first time depression onset in pregnancy. This non-specified poor mood could be an early indicator of sadness, which is a key symptom of depression, though more typically associated with depression occurring among those who are not pregnant or in the postpartum period (Bernstein et al. 2008). Sadness is not conceptualized as feeling “unusually blue” in current screening instruments (Cox et al. 1987; Spitzer et al. 1999) and additional research is needed to understand if feeling ‘unusually blue’ is a precursor or a proxy of early feelings of sadness or is itself a unique risk factor. Lastly, these findings point to identification of a population at particular risk for new-onset depression. The optimal predictive model may be used to direct greater scrutiny for diagnosing and consideration of preventive intervention.

From a health systems and public health standpoint, identifying high-risk but asymptomatic individuals has the potential to change the framework of care delivery through prevention, but also poses challenges in referrals and resource utilization in the face of significant shortages in mental health services (Butryn et al. 2017). Future investigation should focus on approaches to address depression-related risk factors (e.g., hunger prevention, provision of social support, sleep optimization), as well as strategies to prevent or mitigate the onset and effects of depression, while also considering health care utilization and availability of resources. Perinatal mental health providers (e.g., psychiatrists, psychologists, pharmacists, and/or OBGYNs) should consider incorporating these predictive modeling tools into electronic medical records and clinical workflows in a way that is least impeding to front-line staff. Our best performing model used a small number of variables that can be inputted by patients, allowing for ease in implementation in routine care.

### Limitations

One primary limitation to this study is participation bias as data were limited to those patients in our healthcare system who were willing to engage with a pregnancy app prescribed by their obstetrical provider and were willing to self-screen for depression at least once throughout their use. Although the majority of women of reproductive age have smartphones (Pew Research Center 2021) and the majority of pregnant people do use pregnancy apps during their pregnancy (Lee and Moon 2016), the risk factors present for the patient population in our dataset may not be generalizable to all pregnant individuals. In particular, populations that tend to have lower access to the internet and smartphone capabilities also tend to have less access to healthcare resources more generally (Eyrich et al. 2021; Kan et al. 2022; Roberts and Mehrotra 2020). Moreover, while the demographic distribution of individuals included in our dataset is reflective of the greater patient population of our healthcare system, there is limited representation from racially diverse groups that are not well-represented in our regional geography. Similarly, our sample showed some response bias. All participants completed baseline screening, but only 60% volunteered information about health-related social needs, whether due to stigma around disclosure of these issues or limited engagement with the app. As a result, food insecurity - a key variable for predicting new depression – was inconsistently measured across the sample.

To focus on prediction of first-time depression onset in pregnancy, we chose to limit data to those who had no history of depression. We did, however, include those with a history of anxiety, as it is diagnostically and symptomatically distinct from depression and is an established depression risk factor. This decision allowed us to consider anxiety history, which may be missed as a risk factor when not concurrent with depression. Furthermore, although anxiety symptoms often co-occurs with depression symptoms, over 40% of individuals experiencing increasing depression do not experience increasing anxiety (Korja et al. 2018). Participants in this study were not asked to disclose use of specific medications, so it is possible some individuals with a history of anxiety might have been taking antidepressants as part of their treatment. Antidepressant use, not captured by our analysis, could introduce noise in our specificity signal. If this were the case, false-positive predictions would be more likely to occur among those who would otherwise be experiencing depression if not already using medication. Future work will incorporate prescription medication use, among other treatments, to improve the specificity of the screening model.

Lastly, while our analysis includes a consideration of the trade-off of accuracy and simplicity, we do not explicitly measure the interpretability of this algorithm to those individuals who would implement it as a screening approach. In planned future work, we will refine this algorithm in partnership with clinical research partners, such as perinatal mental health care providers and administrators, as well as with perinatal individuals, incorporating their perspectives on the relevance of the questions for screening purposes, discussion of what may be missing, and any potential implementation barriers (Jeyaraman et al. 2023).

## Conclusions

This work demonstrates an approach to developing a machine learning risk prediction model, built from patient-reported data, that can be used in the first trimester to identify those individuals likely to develop first time moderate-severe depression later in their pregnancy. The variables selected by the best-performing model encompass a simple set of questions that could be administered to patients early in pregnancy to support tailored resource provision and early intervention for depression risk. The model also identified some novel pregnancy-specific stressors that may play a unique role in depression risk during the perinatal period and underscore the importance of considering the psychosocial and environmental context in which an individual is navigating their pregnancy.

## Supplementary Material

Online Resource 1

Online Resource 3

Online Resource 2

## Figures and Tables

**Table 1:** Demographics of prenatal patient cohort

Demographics	Study Sample (*n* = 944)	Participants with no depression onset (*n* = 834)	Participants with first time depression (*n* = 110)	*p*-value^1^
**Age**	30 (5.4)	30 (5.4)	28 (5.5)	< 0.01
**Income**				< 0.01
< 25k	166 (17.6%)	123 (14.7%)	43 (39.1%)	
25–50k	165 (17.5%)	146 (17.5%)	19 (17.3%)	
50–100k	310 (32.8%)	284 (34.1%)	26 (23.6%)	
> 100k	268 (28.4%)	249 (29.9%)	19 (17.3%)	
Prefer not to respond	35 (3.7%)	32 (3.8%)	3 (2.7%)	
**Race/Ethnicity**				0.02
Black/African American	113 (12.0%)	89 (10.7%)	24 (21.8%)	
White	734 (77.8%)	661 (79.3%)	73 (66.4%)	
Hispanic	19 (2.0%)	16 (1.9%)	3 (2.7%)	
Asian	35 (3.7%)	33 (4.0%)	2 (1.8%)	
Other	39 (4.1%)	31 (3.7%)	8 (7.3%)	
Prefer not to respond	4 (0.4%)	4 (0.5%)	0 (0.0%)	
**Education status**				< 0.01
< High school or GED	21 (2.2%)	16 (1.9%)	5 (4.5%)	
High school or GED	255 (27.0%)	212 (25.4%)	43 (39.1%)	
Undergraduate	390 (41.3%)	344 (41.2%)	46 (41.8%)	
Postgraduate	271 (28.7%)	255 (30.6%)	16 (14.5%)	
Prefer not to respond	7 (0.7%)	7 (0.8%)	0 (0.0%)	

1 p-values compare baseline characteristics between those with and without first time onset depression in our sample. Age was compared using two-sample t-test, all others compared using χ^2^ test

**Table 2:** Comparison of machine learning models used to predict first time depression in pregnancy using patient self-reported data

Models	AUC [CI]	Sensitivity^i^	Specificity	DOR	RMSE	No. of Variables Retained
PC-KCI	**0.89 [0.84, 0.94]**	**0.81**	0.83	**20.36**	4.28	**14/53 (26%)** ^ii^
Shallow Decision Trees	0.74 [0.62, 0.84]	0.53	0.79	4.18	5.17	**13/55 (24%)**
Forward Stepwise Selection	0.78 [0.68, 0.87]	0.59	0.78	4.94	4.95	40/55 (73%)
LASSO	0.79 [0.70, 0.88]	0.59	0.78	4.94	4.81	45/55 (82%)
Random-Forest	0.78 [0.68, 0.88]	0.35	**0.95**	9.45	**3.94**	55/55 (100%)
XGBoost	0.80 [0.71, 0.88]	0.59	0.79	5.50	4.63	55/55 (100%)

i Predicted score threshold of 11 was chosen to achieve at least 0.80 sensitivity and specificity within the highest performing baseline model for sensitivity, specificity, and DOR

ii PC-KCI is the only model that automatically drops variables in the training set that show no variation, reducing the denominator

**Table 3:** Specific questions retained by the PC-KCI model when health-related social needs are included

Variable Category	Variable	Question Wording
Personal Medical History	*History of Anxiety*	“Do you have a history of diagnosed anxiety?”
Pregnancy-Specific Stressors	*Labor and Delivery Stress*	“Have you recently felt unusually stressed about going into labor and giving birth?”
	*Physical Appearance Stress*	“Have you recently felt unusually stressed about how this pregnancy will affect your health or appearance?”
	*Health Issues Stress*	“Are you feeling bothered, upset or worried at this point in your pregnancy about the effect of ongoing health problems (such as high blood pressure or diabetes) on your pregnancy?”
	*Interpersonal Relationship Stress*	“Have you recently felt unusually stressed about how the new baby will affect your relationships?”
Psychosocial Factors	*Feeling ‘Blue’*	“Since being pregnant, have you been feeling more stressed or blue than usual?”
	*Childhood Emotional Neglect*	“How true are the following statements regarding your upbringing?” • *There was someone in my family who helped me feel important or special.* • *People in my family looked out for each other.* • *I felt loved.*
	*Financial Stress*	“Have you recently felt unusually stressed about your financial stability?”
Health-related Social Needs	*Food Insecurity*	“Within the last 12 months, have you worried you would run out of food, or did you run out of food, before being able to buy more?”

## Footnotes

1 Although the vast majority of EPDS screenings occurred in the 2nd or 3rd trimester, 2.8% of maximum EPDS scores did occur late in the first trimester (i.e., at 11 or 12 weeks). These individuals were not removed from analyses, as sensitivity analyses conducted by removing these individuals show no change in AUCs.
