# Correction to: Predicting first time depression onset in pregnancy: applying machine learning methods to patient-reported data

**DOI:** 10.1007/s00737-025-01572-3

**Published:** 2025-03-01

**Authors:** Tamar Krishnamurti, Samantha Rodriguez, Bryan Wilder, Priya Gopalan, Hyagriv N. Simhan

**Affiliations:** 1 Division of General Internal Medicine, University of Pittsburgh, 230 McKee Pl, Suite 600, Pittsburgh, PA 15213 USA; 2 Machine Learning Department, Carnegie Mellon University, Pittsburgh, PA 15213 USA; 3 UPMC Western Psychiatric Hospital, Pittsburgh, PA 15213 USA; 4 Department of OB-GYN and Reproductive Sciences, University of Pittsburgh, Pittsburgh, PA 15213 USA

**Correction to: Archives of Women’s Mental Health (2024) 27:1019-1031**

10.1007/s00737-024-01474-w

In the originally published article entitled ‘Predicting first time depression onset in pregnancy: applying machine learning methods to patient-reported data’, the reviewer comments and responses to reviewers were incorrectly published by the journal as a supplemental file and the original supplemental files were not published online as intended. The current published version includes the 3 supplemental files referenced in the article.

The original article has been corrected.

