# Supplementary material for: Predicting first time depression onset in pregnancy: applying machine learning methods to patient-reported data
Source: Arch Womens Ment Health. Author manuscript; Available in PMC 2025 Sep 10. (PMC11579171; doi:10.1007/s00737-024-01474-w)
Supplement: Online Resource 3 [file NIHMS2000796-supplement-Online_Resource_3.docx]

**Online Resource 3** Comparison of machine learning models used to predict first time depression from patient self-reported data when health-related social needs are included

| **Measurements** | **AUROC [CI]** | **Sensitivity** | **Specificity** | **DOR** | **Root Mean Squared Error** | **No. Selected/No. of Variables (%)** |
| --- | --- | --- | --- | --- | --- | --- |
| PC-KCI | **0.93  [0.87,0.98]** | 0.90 | 0.81 | 38.50 | 4.71 | **9/56 (16%)^1^** |
| Shallow Decision Trees | 0.92 [0.85,0.97] | **1.00** | 0.74 | **57.64^2^** | 4.67 | 14/60 (23%) |
| Forward Stepwise Selection | 0.75  [0.50,0.95] | 0.60 | 0.86 | 9.38 | 5.57 | 47/60 (78%) |
| LASSO | 0.76 [0.56,0.91] | 0.50 | 0.89 | 7.70 | 4.68 | 39/60 (65%) |
| Random-Forest | **0.93 [0.87,0.98]** | 0.20 | **0.98** | 10.63 | **3.82** | 60/60  (100%) |
| Extreme Gradient Boosting | 0.85 [0.73,0.95] | 0.60 | 0.93 | 20.25 | 4.24 | 60/60 (100%) |

^1^ PC-KCI is the only model that automatically drops variables with no variation in the training set, reducing the denominator.

^2^ Due to no false-negative cases with Shallow Decision Trees, 0.5 was added to the false-positive, false-negative, true-positive, and true-negative totals, introducing bias when calculating the DOR.
