# Supplementary material for: Predicting first time depression onset in pregnancy: applying machine learning methods to patient-reported data
Source: Arch Womens Ment Health. 2024 May 22;27(6):1019–31. doi: 10.1007/s00737-024-01474-w (PMC11579171; doi:10.1007/s00737-024-01474-w)
Supplement: Supplementary file 1 [file 737_2024_1474_MOESM1_ESM.docx]

**Online Resource 1** All pairwise DeLong’s test for differences in AUCs among machine learning models used to predict first time depression from patient self-reported data

| **Model A** | **Model B** | **Including Health-Related Social Needs** | **Alternative Hypothesis** | **DeLong’s Test for Difference in AUC** |
| --- | --- | --- | --- | --- |
| PC-KCI | Shallow Decision Trees | - | A > B | **<0.01** |
| PC-KCI | Shallow Decision Trees | ✓ | A > B | 0.34 |
| PC-KCI | Forward Stepwise Selection | - | A > B | **0.02** |
| PC-KCI | Forward Stepwise Selection | ✓ | A > B | 0.08 |
| PC-KCI | LASSO | - | A > B | **0.03** |
| PC-KCI | LASSO | ✓ | A > B | **0.04** |
| PC-KCI | Random-Forest | - | A > B | **0.03** |
| PC-KCI | Random-Forest | ✓ | A ≠ B | 0.97 |
| PC-KCI | XGBoost | - | A > B | **0.04** |
| PC-KCI | XGBoost | ✓ | A > B | 0.11 |
| Shallow Decision Trees | Forward Stepwise Selection | - | A < B | 0.18 |
| Shallow Decision Trees | Forward Stepwise Selection | ✓ | A > B | 0.09 |
| Shallow Decision Trees | LASSO | - | A < B | 0.25 |
| Shallow Decision Trees | LASSO | ✓ | A > B | 0.06 |
| Shallow Decision Trees | Random-Forest | - | A < B | 0.14 |
| Shallow Decision Trees | Random-Forest | ✓ | A < B | 0.32 |
| Shallow Decision Trees | XGBoost | - | A < B | **<0.01** |
| Shallow Decision Trees | XGBoost | ✓ | A > B | 0.13 |
| Forward Stepwise Selection | LASSO | - | A < B | 0.42 |
| Forward Stepwise Selection | LASSO | ✓ | A < B | 0.47 |
| Forward Stepwise Selection | Random-Forest | - | A ≠ B | 0.76 |
| Forward Stepwise Selection | Random-Forest | ✓ | A < B | **0.05** |
| Forward Stepwise Selection | XGBoost | - | A < B | 0.24 |
| Forward Stepwise Selection | XGBoost | ✓ | A < B | 0.18 |
| LASSO | Random-Forest | - | A > B | 0.46 |
| LASSO | Random-Forest | ✓ | A < B | **0.04** |
| LASSO | XGBoost | - | A < B | 0.43 |
| LASSO | XGBoost | ✓ | A < B | 0.21 |
| Random-Forest | XGBoost | - | A > B | 0.71 |
| Random-Forest | XGBoost | ✓ | A > B | **0.04** |
