# Supplementary material for: Predicting first time depression onset in pregnancy: applying machine learning methods to patient-reported data
Source: Arch Womens Ment Health. 2024 May 22;27(6):1019–31. doi: 10.1007/s00737-024-01474-w (PMC11579171; doi:10.1007/s00737-024-01474-w)
Supplement: Supplementary file 2 [file 737_2024_1474_MOESM2_ESM.docx]

**Online Resource 2** Individual items from each category of variables in the original dataset that were retained across the majority of models

| **Category** | **Variables Retained Across Multiple Models** | **Number of Models Retaining the Variable** |
| --- | --- | --- |
| Demographics | Household income | 6/6 |
|  | Relationship status | 6/6 |
|  | Maternal Race/Ethnicity | 5/6 |
|  | Paternal Race/Ethnicity | 5/6 |
|  | Education | 5/6 |
|  | Age | 5/6 |
| Family Medical History | Preeclampsia | 5/6 |
|  | Diabetes | 4/6 |
|  | Multiples | 4/6 |
| Personal Medical History | Anxiety | 6/6 |
|  | Sleep | 5/6 |
|  | Planned pregnancy | 5/6 |
|  | BMI | 5/6 |
|  | Hypertension | 4/6 |
|  | Diabetes | 4/6 |
| Pregnancy History | Pregnancy Loss | 5/6 |
|  | Miscarriage | 4/6 |
|  | Stillbirth | 4/6 |
|  | NICU admission | 4/6 |
|  | Multiparous | 4/6 |
|  | PPROM | 4/6 |
|  | GDM | 4/6 |
|  | Hypertension | 4/6 |
| Pregnancy-specific Stressors | Health and Appearance | 6/6 |
| Psychosocial Factors | Feeling Blue | 6/6 |
|  | Financial Stressors | 6/6 |
|  | Childhood trauma | 6/6 |
| Current Substance Use | Cigarettes or other tobacco | 5/6 |
|  | Marijuana | 5/6 |
|  | E-cigarettes/vaping | 4/6 |
|  | Benzodiazepines | 4/6 |
